# Supplementary material for: Novel Insights into miR-944 in Cancer
Source: Cancers (Basel). 2022 Aug 31;14(17):4232. doi: 10.3390/cancers14174232 (PMC9454979; doi:10.3390/cancers14174232)
Supplement: Supplementary file 1 [file cancers-14-04232-s001.zip › cancers-1866930-supplementary.pdf]

Supplementary materials

Comparison of miR-944 expression between males and females

The gender information of the patients was obtained from the TCGA database (https://portal.gdc.cancer.gov/), and the Wilcoxon test was used to calculate the significance of the difference in miR-944 between male and female patients. As shown in Figure S1, miR-944 expression levels were not significantly different between males and females in cancers.

Comparison of miR-944 expression between whites and other races

The racial information of the patients was obtained from the TCGA database (https://portal.gdc.cancer.gov/). The patients were divided into white and other races. the Wilcoxon test was used to calculate the significance of differences in miR-944 between whites and other races, and Bonferroni-adjustment by 32 cancer types was used. As shown in Figure S1, we found that in BLCA and ESCA, whites had significantly lower levels of miR-944 than other races.

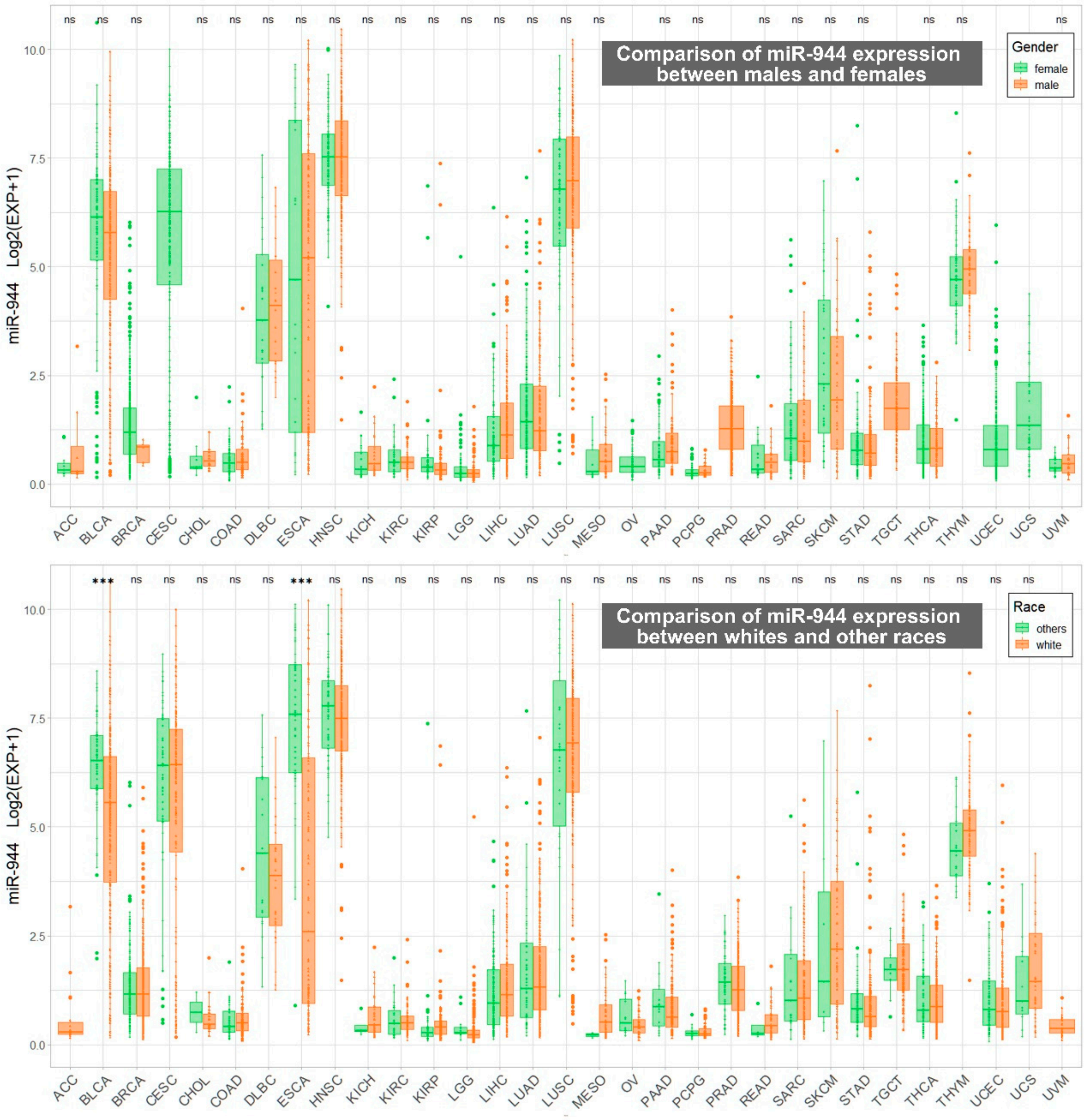

Figure S1. Comparison of miR-944 expression between patients of different gender or race. *** means $p<0.001$; ns means no significant difference ($p>0.05$). ACC, adrenocortical carcinoma; BLCA, bladder urothelial carcinoma; BRCA, breast invasive carcinoma; CESC, cervical squamous cell carcinoma and endocervical adenocarcinoma; CHOL, cholangiocarcinoma; COAD, colon adenocarcinoma; DLBC, lymphoid neoplasm diffuse large B-cell lymphoma; ESCA, esophageal carcinoma; HNSC, head and neck squamous cell carcinoma; KICH, kidney chromophobe; KIRC, kidney renal clear cell carcinoma; KIRP, kidney renal papillary cell carcinoma; LGG, brain lower grade glioma; LIHC, liver hepatocellular carcinoma; LUAD, lung adenocarcinoma; LUSC, lung squamous cell carcinoma; MESO, mesothelioma; OV, ovarian serous cystadenocarcinoma; PAAD, pancreatic adenocarcinoma; PCPG, pheochromocytoma and paraganglioma; PRAD, prostate adenocarcinoma; READ, rectum adenocarcinoma; SARC, sarcoma; STAD, stomach adenocarcinoma; SKCM, skin cutaneous melanoma; TGCT, testicular germ cell tumor; THCA, thyroid carcinoma; THYM, thymoma; UCEC, uterine corpus endometrial carcinoma; UCS, uterine carcinosarcoma; UVM, uveal melanoma.

Association of smoking levels with miR-944

The smoking status information of the patients was obtained from the TCGA database (https://portal.gdc.cancer.gov/), and the patients' smoking index (cigarettes per day × years smoked) was calculated. We further used the smoking index to divide patients into three groups, the non-smoking group (smoking index = 0), the light smoking group (smoking index <200), and the moderate-to-heavy smoking group (smoking index ≥ 200). The Wilcoxon test was used for the significant difference analysis between pairs, and the Kruskal test was used for the different tests of multiple groups of samples. The results were shown in Figure S2. In LUAD, the expression level of miR-944 in light smoking patients was significantly lower than that in non-smoking patients.

Association of alcohol history with miR-944

The patients' drinking history information was obtained from the TCGA database (https://portal.gdc.cancer.gov/), and the patients were divided into two groups: those with a drinking history and those without a drinking history. The results were shown in Figure S2, there was no significant difference in the level of miR-944 between the two groups of patients, indicating that drinking history did not affect the expression level of miR-944 in patients.

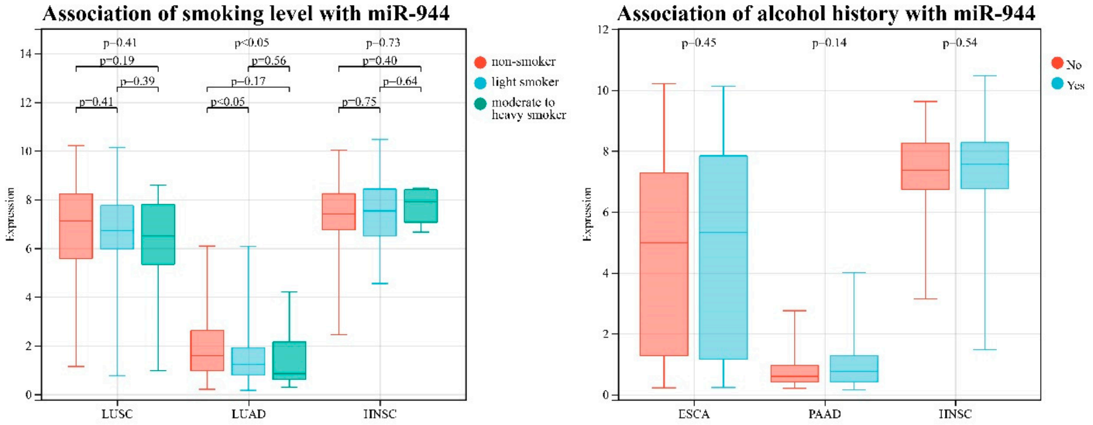

Figure S2. Effect of smoking and alcohol history on miR-944 expression in patients. ESCA, esophageal carcinoma; HNSC, head and neck squamous cell carcinoma; LUAD, lung adenocarcinoma; LUSC, lung squamous cell carcinoma; PAAD, pancreatic adenocarcinoma.

Association of overall survival (OS) with miR-944

We obtained the difference in OS levels between miR-944 high and miR-944 low expression groups from CancerMIRNome (http://bioinfo.jialab-ucr.org/CancerMIRNome/) [PMID: 34500460]. The results were shown in Figure S3. In BLCA, patients with high expression of miR-944 had better OS, while patients with high expression of miR-944 in LGG and THYM had poor prognoses ($p<0.05$).

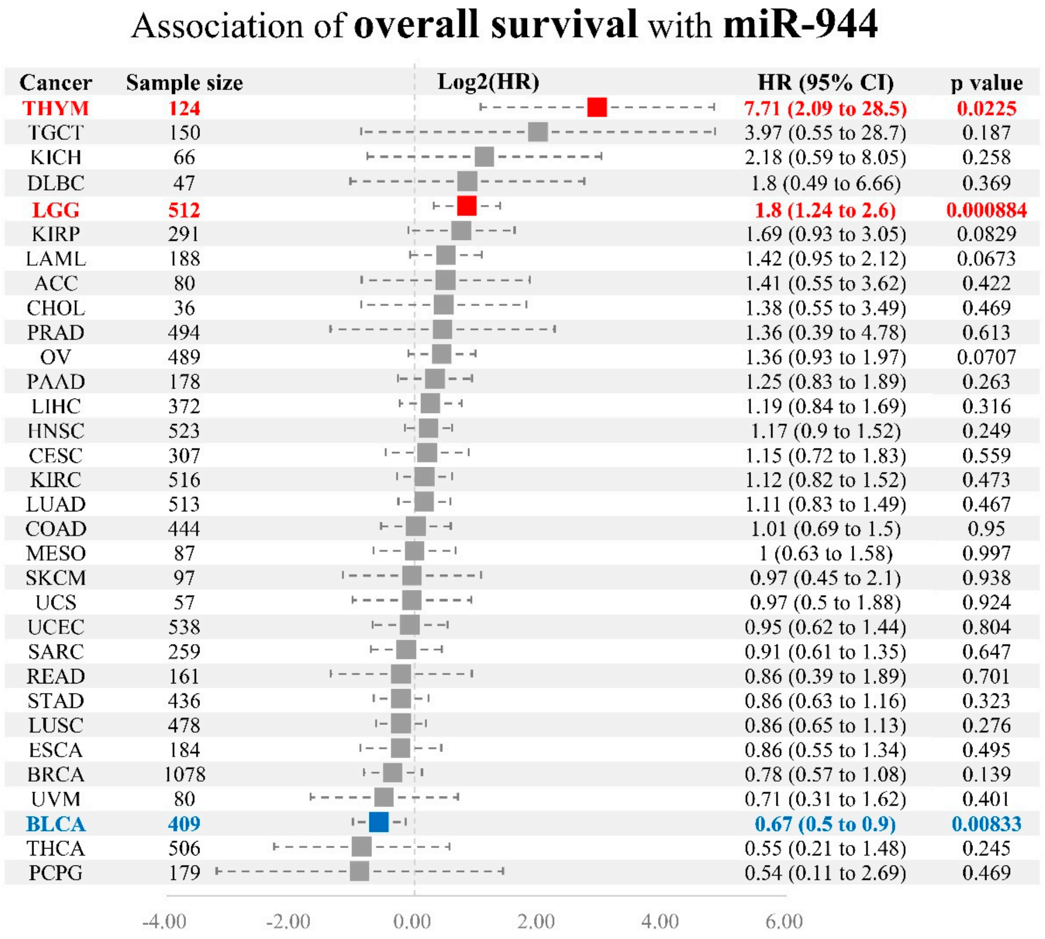

Figure S3. Association of OS with miR-944 in pan-cancer based on TCGA database. The blue font indicates that miR-944 is significantly high expressed with longer OS of patients in this cancer type ($p<0.05$); the red font indicates that miR-944 is significantly high expressed with shorter OS of patients in this cancer type ($p<0.05$); the gray font indicates that miR-944 is not significantly expressed with OS of patients in this cancer type ($p>0.05$). ACC, adrenocortical carcinoma; BLCA, bladder urothelial carcinoma; BRCA, breast invasive carcinoma; CESC, cervical squamous cell carcinoma and endocervical adenocarcinoma; CHOL, cholangiocarcinoma; COAD, colon adenocarcinoma; DLBC, lymphoid neoplasm diffuse large B-cell lymphoma; ESCA, esophageal carcinoma; HNSC, head and neck squamous cell carcinoma; KICH, kidney chromophobe; KIRC, kidney renal clear cell carcinoma; KIRP, kidney renal papillary cell carcinoma; LAML, Acute Myeloid Leukemia ;LGG, brain lower grade glioma; LIHC, liver hepatocellular carcinoma; LUAD, lung adenocarcinoma; LUSC, lung squamous cell carcinoma; MESO, mesothelioma; OV,

ovarian serous cystadenocarcinoma; PAAD, pancreatic adenocarcinoma; PCPG, pheochromocytoma and paraganglioma; PRAD, prostate adenocarcinoma; READ, rectum adenocarcinoma; SARC, sarcoma; STAD, stomach adenocarcinoma; SKCM, skin cutaneous melanoma; TGCT, testicular germ cell tumor; THCA, thyroid carcinoma; THYM, thymoma; UCEC, uterine corpus endometrial carcinoma; UCS, uterine carcinosarcoma; UVM, uveal melanoma.
